# Mathematical model of ammonium nitrogen transport with overland flow on a slope after polyacrylamide application

**DOI:** 10.1038/s41598-018-24819-9

**Published:** 2018-04-23

**Authors:** Chang Ao, Peiling Yang, Shumei Ren, Weimin Xing

**Affiliations:** College of Water Resources and Civil Engineering, China Agricultural University, Beijing, 100083 China

## Abstract

The nutrient loss caused by soil erosion is the main reason for soil degradation and environmental pollution, and polyacrylamide (PAM) as a common soil amendment has a great influence on runoff and erosion processes at the slope. In order to investigate the mechanism of nutrient transport with runoff, a field experiment was conducted and a simple mathematical model was developed in this study. Four PAM application rates (0, 1, 2, and 4 g·m^−2^) and two rainfall intensities (50 and 80 mm·h^−1^) were applied in the field experiment. The results revealed that runoff rate of 2 g·m^−2^ PAM application treatments decreased by 5.3%-10.6% compared with the control groups, but it increased by10.9%-18.7% at 4 g·m^−2^ PAM application treatments. Polyacrylamide application reduced ammonium nitrogen concentrations of runoff by 10.0% to 44.3% relative to the control groups. The best performance with correlation coefficient (*R*^2^) and Nash–Sutcliffe efficiency (*NSE*) showed that the ammonium transport with runoff could be well described by the proposed model. Furthermore, the model parameter of the depth of the mixing layer (*hm*) linearly increased with an increase in flow velocity, but exponentially decreased with an increase in PAM application rate.

## Introduction

Ammonia nitrogen is one of the necessary nutrients used for agriculture crops. However, the ammonia nitrogen losses associated with erosion can threaten the quality of sloping field and healthy of surface water. The surface runoff is one of the most important pathways for nutrient transport in a slope field^1–3^. Soil nutrient transport via surface runoff is a complex process that is affected by many factors such as rainfall characteristics, soil physics, chemical properties, slope gradient, slope length and surface coverage^4^. Numerous studies have been carried out to describe this process of solute transport through physical or mathematical models^5–14^. Based on their respective assumptions, the models of solute transport were generally divided into three categories: empirical models, mixing-layer models and interfacial diffusion-controlled models^15^.

Empirical models describe the transfer process by a principle similar to the Universal Soil Loss Equation(USLE)^16^. The major influencing factors of this model are the soil, slope and rainfall characteristics. These models have simple equations and are easy to compute, but they usually require large experimental data for parameter fitting. Mixing-layer models, which assume that rainwater instantaneously and completely mixes with soil water in a very thin layer (the mixing layer), the solute concentrations of runoff, infiltrating water and soil water are consistent in mixing layers^5–8^.Because of simple formulations and relatively few parameters, the mixing-layer models were widely used in the solute transport processes. But there is currently no effective method to accurately determine the mixing-layer depth. Walter *et al*.^11^ reported that initial moisture content, rainfall intensity, and slope gradient influenced the mixing depth. Yang *et al*.^17^ found that the mixing depthincreased as rainfall intensity, slope gradient, and initial water content increased. Yang *et al*.^4,18^ later found that the depth of the mixing layer was a function of rainfall time and not a constant as described in previous studies. Interfacial diffusion-controlled models consider that the transfer of solutes from soil to runoff is diffusion-driven or dispersed via raindrops^9–13^. The interfacial diffusion-controlled model models are physics-based and include the effect of rainfall dispersion on interfacial process. Wallach and van Genuchten^8^ proposed a convective-dispersive model based on the assumption that the solute transport flux from soil surface to overland flow was driven by diffusion. Later, Zhang *et al*.^14^ proposed a model by coupling mixing zone concept with convective-diffusion equation. Then, Gao *et al*.^10,11^ indicated that the mixing depth was equivalent to shield depth, whereas the solute concentration in the runoff and in the mixing layer was not consistent. As shown above, the mixing-layer or exchange-layer plays a very important role in the mixing-layer models or interfacial diffusion-controlled models. Currently, the values of solute concentration estimated using mixing-layer models and interfacial diffusion-controlled models are accepted by most researchers as reliable “measured values”, and practical applications of related research across the world have produced good results.

The purpose of this paper was to construct a solute transport model for polyacrylamide (PAM)-treated slopes. The key was to study the effects of PAM application on solute transport processes. Polyacrylamide as a soil amendment was frequently used to limit runoff, erosion and nutrient loss^19–28^. Many studies have shown that a PAM application rate of 1–2 g·m^−2^ is most effective on decreasing runoff and soil erosion^24–26^. After PAMs are applied to soil, soil particles and polymers are bridged together by multivalent cations in the soil solution, which increases aggregate stability and prevents seal formation at the soil surface, thereby decreasing soil and nutrient losses. Sojka^27^ found the pollutant concentration of runoff was decreased with PAM application under surface irrigation. In a field experiment, Chen *et al*.^29^ found that PAM application reduced total nitrogen losses by 35.3% to 50.0% and total phosphorus losses by 34.9% to 48.0% relative to the control group. However, solute transport modeling involving PAMs is made quite challenging by their unknown effects on surface soil structure and the mixing depth. Many studies have demonstrated that addition of PAMs was effective in improving surface structure, but excess PAMs could clog soil pores, thereby increasing runoff rate.PAM application reduced erosion rate and flow velocity which play crucial roles in soil nutrient transport^19,22,23^. To predict solute transport after PAM application, a simple model was developed in this study based on the mixing layer models and the interfacial diffusion-controlled models. It is assumed that the exchange rate between nutrient in the soil and overland flow is a constant which may be affected by rainfall intensity and PAM application rate. Similarly, the rainwater instantaneously and completely mixes with soil water in the mixing layer.

Therefore, the objectives of the study were to: (1) investigate the runoff, flow velocity, soil and ammonia nitrogen losses after PAM application, (2) develop a mathematical model to describe ammonia nitrogen transport from soil surface to runoff and estimate the associated model parameters, and (3) explore the relationship between PAM application and mixing layer depth.

## Materials and Methods

### Study area description

**Figure 1:**
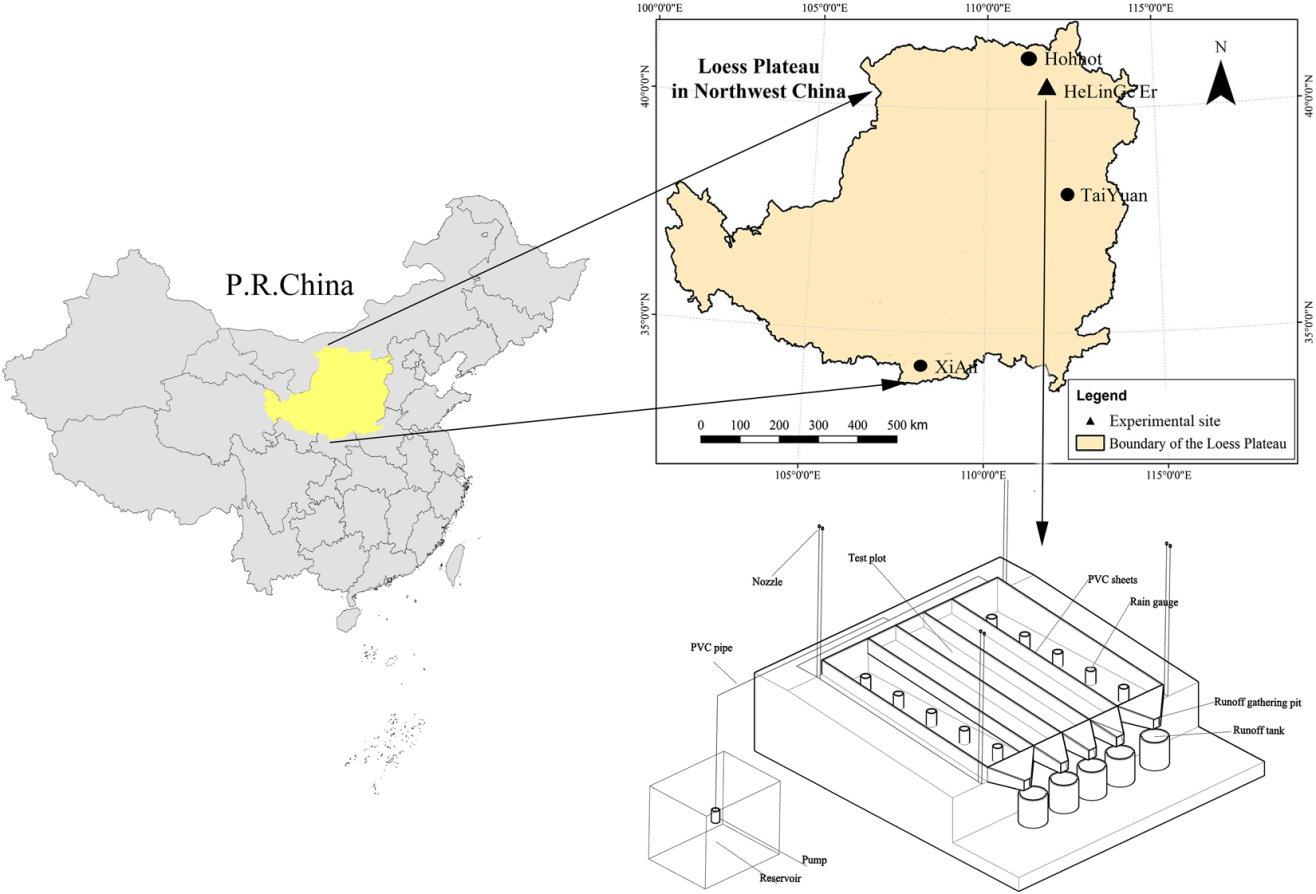
The experimental plot and simulation rainfall system. The map was generated using ArcMap 10.2.2 (http://www.esri.com).

The experiment was carried out on a field slope land (N40°12′, E111°41′) on a loess plateau, 2 km south of the China Agricultural University Hohhot Experimental Station in Hohhot, Inner Mongolia Autonomous Region, China (Fig. 1). The study site has a semi-arid climate with a mean annual temperature of 6.2 °C and annual precipitation of 417.5 mm. Most of the precipitation occurs from June to September, and the maximum recorded 24-hour precipitation is 99 mm. The soil is classified as Kastanozem with a sandy texture. The partition coefficients of sand, silt and clay were 89.6%, 5.4% 5.0%, respectively. The soil is susceptible to erosion. The mean bulk density for the surface 30 cm of soil was 1.45 g·cm^−3^. The concentrations of organic matter and total nitrogen were 2.81 and 0.17 g·kg^−1^. The pH of soil was 8.4.

### Polymer and Rainfall simulation

Polyacrylamide samples, which were provided by Beijing Chemical Ltd., Beijing, China, were used in the experiment. The active ingredient concentration of polyacrylamide was 99.9%, and the samples were white powder particles with a diameter of <0.02 mm. Polyacrylamide was anionic, and the molecular weight was 1.2×10^6^ g·mol^−1^. These data were obtained from the chemical company.

Field rainfall tests were carried out via an artificial rainfall simulation in field runoff plots. The artificial rainfall simulation was designed and manufactured by the college of water conservancy and civil engineering in the China agricultural university. The rainfall simulator consisted of eight nozzles and a water supply system. The simulator nozzles were 3.2 m above the soil surface with 90-degree spray angle at the four corners of the runoff plots. The effective rainfall area was 5.0 m × 5.0 m. The range of rainfall intensity was 20–120 mm·h^−1^. The uniformity was above 85%. The measured median raindrop diameter was 1.5 mm, and the calculated kinetic energy of rainfall was 15.82 J·mm^−1^·m^−2^. The local groundwater was used as simulated rainfall water. The CO_3_^2−^, HCO_3_^−^, Cl^−^, SO_4_^2−^, Ca^2+^, Mg^2+^, K^+^ and Na^+^ concentrations of the local groundwater were 3.4, 215.5, 44.8, 58.3, 82.9, 31.3, 2.36 and 44.5 mg·L^−1^, respectively. And the pH of the local groundwater was 7.61.

### Experimental setup

Experimental treatments in the study included four PAM application rates (0, 1.0, 2.0, and 4.0 g·m^−2^) and two rainfall intensities (50 and 80 mm·h^−1^). All the simulated rainfall durations were 45 minutes. Therefore, the rainfall amount was 37.5 mm for a 50 mm·h^−1^ rainfall intensity rate and 60 mm for an 80 mm·h^−1^ rainfall intensity rate. The experiment contained eight treatments, and 3 replications were performed for each treatment. The plot area was 5 m × 5 m and equal to the effective rainfall area (Fig. 1). The slope gradient of the plot was 5 degrees. The intermediate region had a width of 2.4 m and was selected as the test area. Five rain cylinders were evenly placed on both sides of the test area to calibrate rainfall intensity during the simulated rainfall (Fig. 1). The intermediate region was equally divided into three sub-districts by plastic plates. The sub-district was 0.8 m wide and 5 m long. In addition, 0.3 m plastic plates were buried in the soil to separate the infiltration, and the other 0.3 m plastic plates were placed above the soil to separate runoff.

The test district was weeded and leveled as much as possible with a shovel and then remained inactive for approximately two weeks to allow the soil to consolidate. Carbamide, the most commonly used fertilizer in the study site as nitrogen fertilizer, was dissolved and sprayed on the soil surface with an application rate of 50 g·m^−2^. The N fertilization rate of used carbamide was 46.3%. Then, mixtures of PAMs and 10 kg air dried soil were uniformly spread on the slope surface during windless times. Tests were carried out around five o ‘clock in the morning to eliminate the effects of wind. To ensure that the initial moisture content of each treatment was consistent, the slope was pre - wet with rainfall intensity of 20 mm·h^−1^ 12 hours until the runoff initially formed before the tests started. The surface soil was collected before the simulated rainfall to measure the adsorption of soil mixing with PAM and the initial nutrient concentration in the soil.

The time that the runoff initially formed was recorded for each rainfall event, and each rainfall simulation time lasted for 45 minutes. Runoff samples were collected at the outlet of the flume in a plastic bucket at unequal intervals: 0–1 min after the runoff initially formed and 10–11 min, 15–16 min, 20–21 min, 30–31 min, 40–41 min and 45–46 min of the rainfall time. Runoff samples were separated with a filter membrane to obtain runoff and sediments. The runoff was weighed to calculate the volume and sediments on the filter membrane were weighed after they were oven-dried. The velocity of the slope flow was measured by the dye tracer (red ink) method^30^.

The viscosities of the runoff were measured with a rotary viscometer (NDJ-9s) which were provided by Lichen Ltd., Shanghai, China. A 25-ml sample of the runoff (or local groundwater) was introduced into a beaker, which was then placed under the viscometer. The rotor of the viscometer must be completely immersed in the solution and the temperature of solution must be constantly 25 °C via the constant temperature slot. Then, the instrument was activated, and the viscosity was recorded. The relative viscosity of runoff was calculated by^17^

1 $${\rm{\eta }}={\eta }_{r}/{\eta }_{lg},$$

Where ${\rm{\eta }}$ is the relative viscosity; ${\eta }_{r}$ is the viscosity of the runoff (mpa·s); and ${\eta }_{r}$ is the viscosity of local groundwater (mpa·s).

All runoff samples after filtration were stored at 0–4 °C in a refrigerator for nutrient analysis. The concentrations of ammonium were determined by a continuous Flowing Analyzer (Alliance Futura) at 660 wave lengths. The adsorption of the soil mixture and PAM was measured by isothermal adsorption test. The isothermal adsorption test was carried out using the same method with Olsen^31^ and Chen^32^.

### Theoretical analysis

The transportation of dissolved nutrients from soil surface to runoff is the focus of this study. Based on the concept of diffusion-based models^9,10^ and the complete mixing model^5^, where the solute in runoff only comes from the mixing depth, the solute mass conservation of the mixing layer can be expressed as follows:

2 $$\frac{d({h}_{m}{c}_{m}({\theta }_{s}+{\rho }_{s}k)}{dt}=-\,{k}_{m}{c}_{m}-i{c}_{m},$$

Where ${h}_{m}$ is depth of the mixing layer (m), ${c}_{m}$ is solute concentration in the mixing layer (g·m^−3^), ${\theta }_{s}$ is the saturated moisture content (m^3^·m^−3^), ${\rho }_{s}$ is the soil bulk density (g·m^−3^), $k$ is solute adsorption coefficient (m^3^·g^−1^), $t$ is the rainfall time (s) and $i$ is the infiltration rate (m·s^−1^). Additionally, ${k}_{m}$ is the convective mass transfer coefficient (m·min^−1^) that characterizes the rate of solute transport to the runoff from the mixed layer that depends on rainfall characteristics, soil physics properties, surface coverage and the solute involved in the process.

The Philip’s formula^33^ is often used to simulate the infiltration process in the soil surface. In the early stages of rainfall (before runoff generation), the infiltration rate is equal to the rainfall intensity. As the rainfall continues, the surface soil is gradually compressed by raindrops, and the infiltration rate gradually decreases. When the infiltration rate is less than the rainfall intensity, the surface runoff is formed at the ponding time^14^. Therefore, the infiltration rate can be expressed as follows:

3 $$\{\begin{array}{ll}i=r & t\le {t}_{p}\\ i=0.5S{t}^{-0.5} & t > {t}_{p}\end{array}$$

Where ${t}_{p}$ is the initial runoff time (s) and $S$ is the sorptivity (m·s^−1/2^).

The overland-flow depth on the sloped land is very thin, approximately0.1–0.5 mm^35^. Therefore, the ponding water was neglected, and the runoff can be expressed as follows:

4 $$q(t)=r-i=r-0.5S{t}^{-0.5}t > {t}_{p}$$

Substituting Eq. (3) into Eq. (2), and integrating Eq. (2), and the solute concentration in the mixing layer changing over time can be expressed as follows:

5 $$\,{c}_{m}(t)={c}_{0}\exp (-\frac{{k}_{m}t+S{t}^{0.5}}{{h}_{m}({\theta }_{s}+{\rho }_{s}k)})t > {t}_{p}$$

The solute mass conservation in ponding layer can be expressed as follows:

6 $$q(t){c}_{w}(t)={c}_{m}(t){k}_{m}t > {t}_{p}$$

Substituting Eq. (4) and Eq. (5) into Eq. (6), and the solute concentration in runoff can be expressed as follows:

7 $$\,{c}_{w}(t)={c}_{0}{k}_{m}\exp (-\frac{{k}_{m}t+S{t}^{0.5}}{{h}_{m}({\theta }_{s}+{\rho }_{s}k)})/q(t)$$

### Data analysis

Multivariate nonlinear regression analyses were calculated to estimate the parameters of the proposed runoff model and the nutrient transport model of each simulated rainfall with Matlab 2012. An analysis of variance (ANOVA) method was applied to compare the difference among treatments. For all analyses, p < 0.05 was considered statistically significant. The correlation coefficient (*R*^2^), Nash-Sutcliffe efficiency coefficient (*NSE*) and root mean square error (RMSE) were applied to quantify the agreement between the simulated results and measured data. The *R*^2^, *NSE* and *RMSE* can be expressed as:

8 $${R}^{2}=\frac{{\sum }_{1}^{N}{({o}_{i}-{p}_{i})}^{2}}{{\sum }_{1}^{N}{({p}_{i}-\overline{{p}_{i}})}^{2}}$$

9 $$NSE=1-\frac{{\sum }_{i=1}^{N}{({o}_{i}-{p}_{i})}^{2}}{{\sum }_{i=1}^{N}{({o}_{i}-\overline{o})}^{2}}$$

10 $$RMSE=\sqrt{\frac{{\sum }_{i=1}^{N}{({o}_{i}-{p}_{i})}^{2}}{N}}$$

Where $N$ is the total number of data points, ${o}_{i}$ is the corresponding observed data at point $i$, and ${p}_{i}$ is the simulated value at point $i$, $\bar{{p}_{i}}$ is the average measured value, $\bar{o}$ is the average simulated value.

## Results and Discussion

### Runoff rate

**Figure 2:**
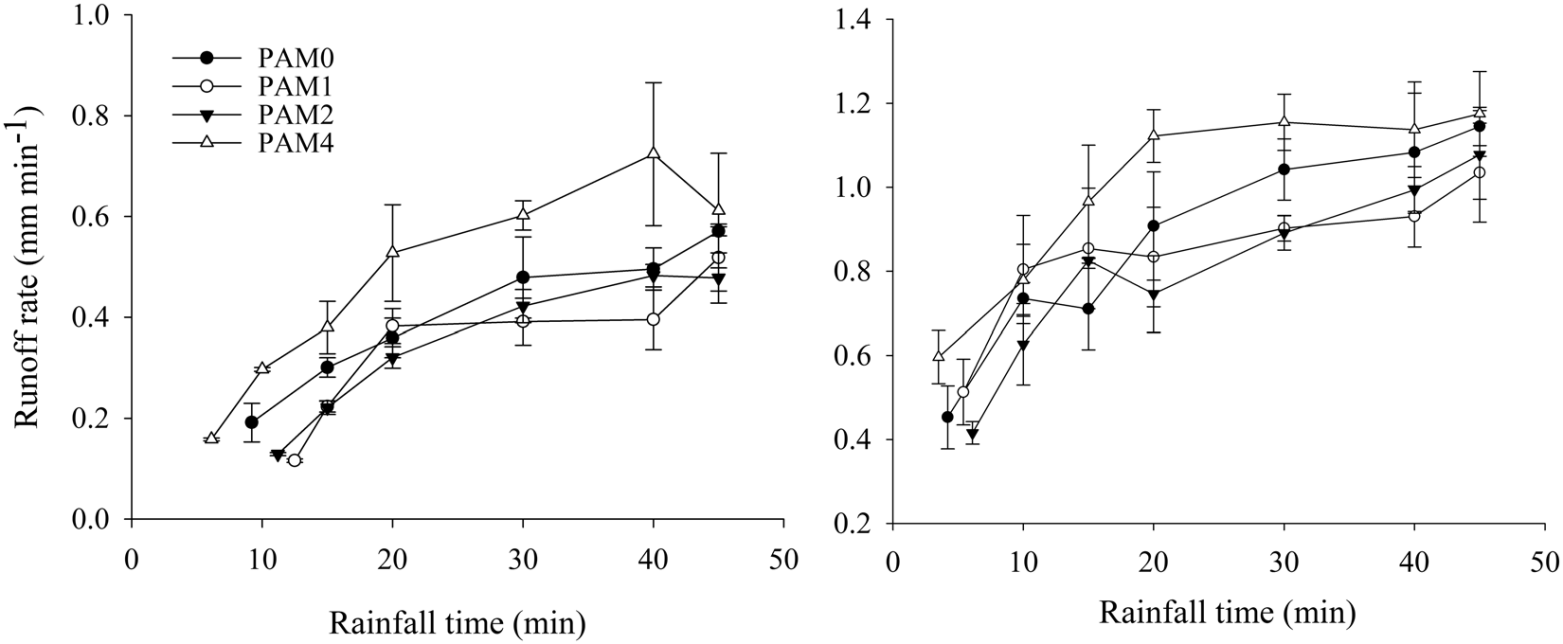
Mean runoff rates over rainfall time for the various treatments (**a**) r = 50 mm·h^−1^, (**b**) r = 80 mm·h^−1^.

The runoff rate for the 4 m^2^ plots with a steepness of 5° under rainfall intensities of 50 and 80 mm·h^−1^ were shown in Fig. 2. The runoff rates gradually increased with rainfall duration (Fig. 2). The runoff rates were larger for 80 mm·h^−1^ than 40 mm·h^−1^. The higher rainfall intensity had little impact on the infiltration capacity of the soil, which was determined by the structure of the soil surface^4^. While the amount of rainfall intensity 80 mm·h^−1^ was larger than that of 40 mm·h^−1^. This leads to the larger runoff rates under rainfall intensities of 80 mm·h^−1^. Compared with the control groups, runoff rates were slightly reduced when the PAM application rate was 1 or 2 g·m^−2^ but significantly increased in treatments with a PAM application rate of 4 g·m^−2^. The runoff rate of 2 g·m^−2^ PAM application treatments decreased by 5.3%-10.6% compared with the control groups, but it increased by10.9%-18.7% at 4 g m^−2^ PAM application treatments. Similar results were found by Abrol^19^, Yu^24^ and Ao^26^. In the control treatment, crust formation was primarily responsible for the large runoff rates because it reduced the infiltration capacity of surface soil. In the PAM-treated soil, the effect of PAM solution-based adhesion and adsorption on soil is that small soil particles become aggregated, which enhances the overall stability of soil aggregates and limits crust formation at the soil surface, resulting in an increase in the infiltration rate and decrease in runoff. However, excess dissolved PAM could clog soil pores^35–37^, thus forming a sealed layer on the surface soil similar to surface crust resulting from the impact of raindrops. Therefore, runoff rates were reduced with 1 and 2 g·m^−2^ PAM application rates but increased with4 g·m^−2^ PAM application rates.

The runoff rates were described by the proposed runoff model which was based on Philip’s formula. Values of the sorptivity ($S$), root mean square error *(RMSE*), correlation coefficient (*R*^2^) and Nash–Sutcliffe efficiency (*NSE*) obtained from runoff model fitting results were listed in Table 1. The *RMSE*, *R*^2^ and *NSE* values showed that the simulated runoff generally agreed well with the measured data. In addition, the ${S}$ decreased with the increase in rainfall intensity, but first increased and then decreased with the increase in PAM application rates. The $S$ is mainly determined by soil structure and the surface soil seal, which determines the infiltration capacity of the soil. Therefore, these results also indicated that the infiltration capacity increased with 1 and 2 g·m^−2^ PAM application rates and decreased with the 4 g·m^−2^ PAM application rate. Additionally, this result was consistent with the runoff coefficient results (Fig. S1).

**Table 1:** Values of measured rainfall intensity, initial runoff time (*t*_*p*_), $S$, *R*^2^, *RMSE* and *NSE* for all treatments after fitting the experimental data.

Designed rainfall intensity (mm h^−1^)	PAM (g·m^−2^)	Measured rainfall intensity (mm·h^−1^)	*t*_*p*_ (min)	*S* (mm·min^−0.5^)	*R* ^2^	*RMSE*	*NSE*
50	0	52.2	9.2	4.03	0.97	0.029	0.97
	1	51.2	12.5	4.74	0.86	0.024	0.86
	2	48.0	11.2	4.65	0.99	0.024	0.99
	4	53.6	6.1	3.12	0.94	0.068	0.87
80	0	82.7	4.2	3.72	0.91	0.039	0.91
	1	83.0	5.4	3.93	0.90	0.053	0.83
	2	79.3	6.1	4.46	0.93	0.034	0.93
	4	80.2	3.5	2.85	0.92	0.020	0.89

Further regression analysis was calculated to investigate the relationship between $S$, rainfall intensity and the PAM application rate. The regression equation can be expressed as follows:

11 $$S=(\,-\,0.65{R}_{PAM}^{2}-2.09{R}_{PAM}+9.4){r}^{-0.22}\,{R}^{2}=0.96$$

### Relative viscosity and flow velocity

**Figure 3:**
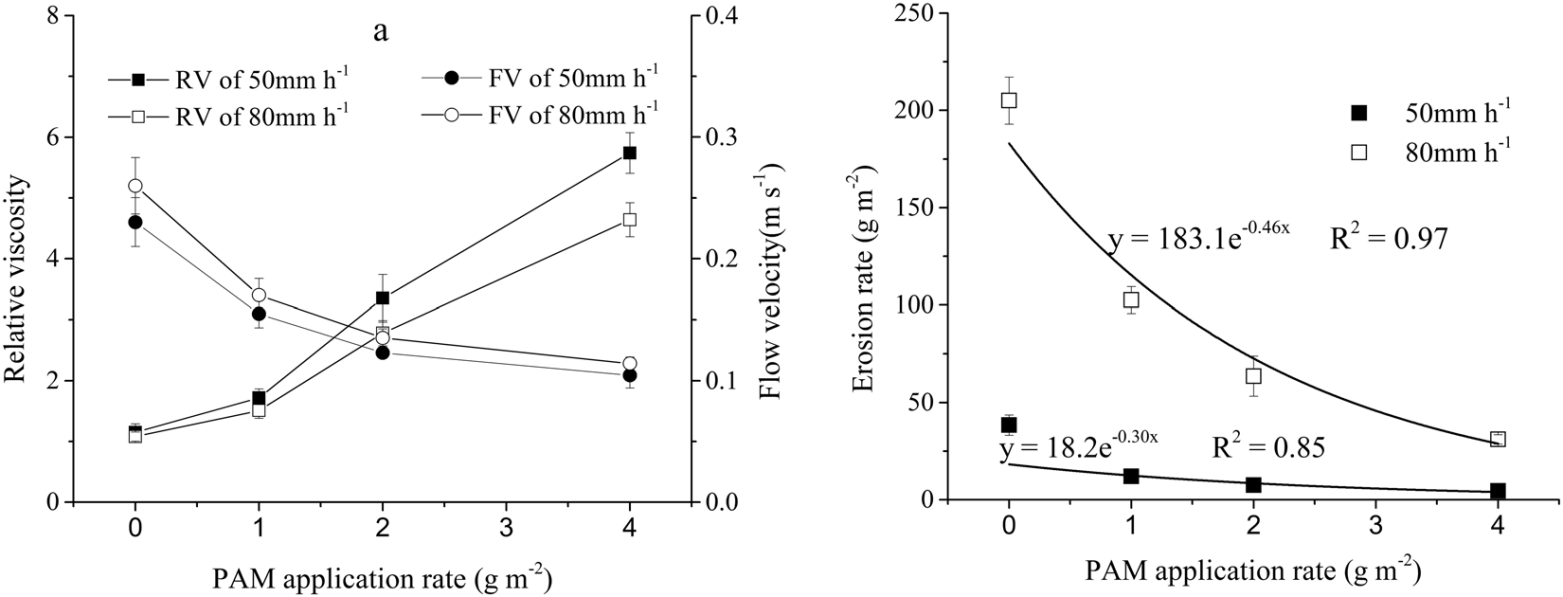
(**a**) Relative viscosity in the runoff and flow velocity of a slope associated with different treatments, error bars = standard deviation. (**b**) The erosion rate as a function of PAM application under 50 and 80 mm·h^−1^ rainfall intensities. Symbols = data; lines = fitted regressions; error bars = standard deviation.

The relative viscosity of the runoff and the flow velocity of the slope for different PAM application rates were shown in Fig. 3. The relative viscosity of runoff increased with the increase in PAM application rates (Fig. 3a). This could be explained by the larger opportunity of PAM loss rate on the slope under larger PAM application rate.

The smallest flow velocities (0.10–0.12 m·s^−1^) were measured in the 4 g·m^−2^ PAM treatments, and the largest flow velocities (0.23–0.26 m·s^−1^) were measured when no PAM treatments were applied (Fig. 3a). Intermediate flow velocities were obtained at intermediate PAM application rates (Fig. 3a). This could be explained by two reasons: one is that PAM dissolved in soil surface increases the cohesion and friction force between runoff and soil; the other is that PAM increases the viscosity of runoff solution, which reduces the velocity of runoff. Therefore, the runoff velocity decreased with the increase of PAM application rate.

### The erosion rate

The erosion rates of different rainfall intensities as a function of PAM application rates were shown in Fig. 3b. The erosion rates of 50 mm·h^−1^ rainfall intensity were smaller than the erosion rates of 80 mm·h^−1^ rainfall intensity (Fig. 3b). Soil erosion generally includes the process of raindrop splashing and scouring of the runoff ^38–40^. The large flow velocity in 80 mm·h^−1^ rainfall intensity increased the shear forces of the runoff, which led to an increased erosion rate.

The erosion rates reduced as the PAM application rate increased under 50 and 80 mm·h^−1^ rainfall intensities (Fig. 3b). The smallest erosion rates were obtained in the 4 g m^−2^ PAM treatments, despite the fact that the runoff rates were the highest in this treatment at the two rainfall intensities. Similar results were obtained by Abrol^19^ and Tang^21^. Several mechanisms may have contributed to the reduction in erosion rates in PAM-treated slopes compared to the control slopes. The increase in viscosity between runoff and surface soil decreased the flow velocity and shear or drag forces that can detach soil particles^41,42^. The dissolved PAM that aggregated small soil particles into larger aggregates^43,44^ were resistant to scouring by the runoff^45,46^. Finally, PAM flocculated soil particles formed a sealed layer on the soil surface. This sealed layer was similar to surface crust resulting from the impact of rain drops, which enhanced the erosion resistance of surface soil.

The relationship between the erosion rates and PAM application rates could be well described by an exponential function (Fig. 3b). The erosion rates increased with rainfall intensities as a power function^40^. Therefore, the sediment delivery rate from soil, $e$, can be written as follows:

12 $$e=71{r}^{4}\exp (\,-\,0.59{R}_{PAM}).$$

### Transport of ammonia nitrogen to the runoff

**Figure 4:**
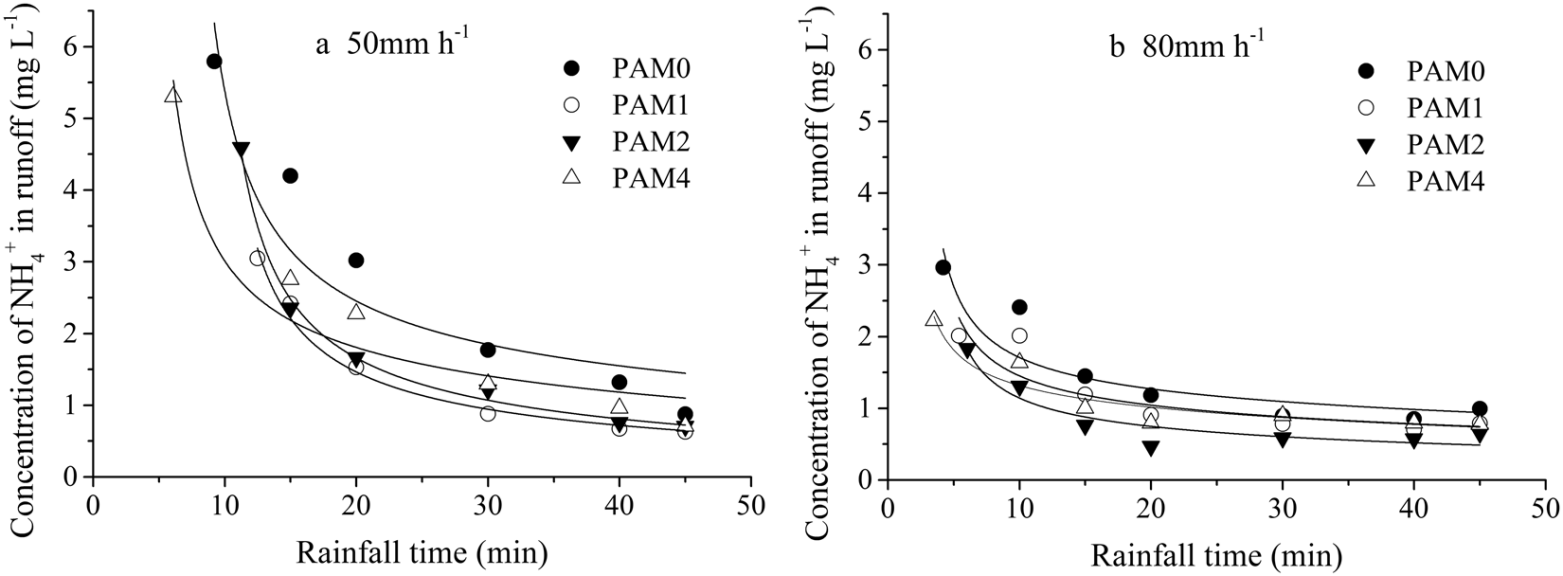
Concentration of NH_4_^+^ in runoff versus rainfall time for different PAM application rates under (**a**) 50 mm·h^−1^ rainfall intensity and (**b**) 80 mm·h^−1^ rainfall intensity.

The ammonium (NH_4_^+^) concentrations as a function of time in runoff were shown in Fig. 4. Ammonium concentration reduced sharply with rainfall time at the initial runoff period, then decreased slowly to a nearly stable value after approximately 20 min (Fig. 4). The NH_4_^+^ concentration in the runoff was mainly controlled by the runoff rate and the NH_4_^+^ concentration in the mixing layer for several minutes after runoff occurred. During the initial runoff stage, the runoff rate increased rapidly, and the NH_4_^+^ concentration in the soil surface gradually reduced with rainfall duration. For these reasons, the NH4 concentration decreased sharply at the runoff source. The NH_4_^+^ concentrations in runoff at the initial runoff period of 50 mm·h^−1^ rainfall intensity were higher compared with that of 80 mm·h^−1^ rainfall intensity. During the experiment, at 80 mm ·h^−1^ rainfall intensity, the nutrient concentration in the runoff reaches stability earlier. This is mainly due to the earlier stabilization of the runoff rate at high rainfall intensities.

The NH_4_^+^ concentrations in runoff decreased with PAM application rates (Fig. 4). Polyacrylamide application reduced ammonium nitrogen concentrations of runoff by 10.0% − 44.3% relative to the control groups. The solute in the runoff mainly contributed to the molecular diffusion and soil erosion of the raindrop splash and scouring during the flow^9–11^. The erosion rates decreased with PAM application on the soil surface (Fig. 3b); therefore, the solute transport from soil to surface runoff decreased after PAM application. Nevertheless, the PAM application reduced the slope flow velocity (Fig. 3a), which increased the contact time between runoff and surface soil. The long contact time led to an increase in the full transport of solutes from soil solution to runoff. Therefore, the ammonium concentrations of 4 g·m^−2^ PAM treatments were greater than that of1 and 2 g·m^−2^ PAM treatments.

The amount of ammonium nitrogen losses in runoff of PAM application rate 0, 1, 2 and 4 g·m^−2^ were 131.34, 52.48, 64.50 and 136.62 mg under rainfall intensity of 50 mm·h^−1^, respectively. It decreased firstly and then increased as PAM application rate increased. This had a similar trend with that of rainfall intensity 80 mm·h^−1^. However, the ammonium nitrogen losses in sediments decreased with an increase of PAM application rate. The runoff-associated nitrogen loss occupied 57–96% of the total nitrogen loss. The percentage of ammonium nitrogen losses in runoff decreased with an increase in rainfall intensity but increased with an increase in PAM application rate. The particles-associated nitrogen loss occupied 3–42% of the ammonium nitrogen losses for a single rainfall event (Table 2). The proportion of the particle-associated nitrogen increased when the rainfall intensity increased. This was mainly caused by the fact the sediment loss increased with an increase in rainfall intensity. The particle-associated nitrogen loss decreased with the increase of PAM application rates. This was closely related with the decreasing trend of sediment yield with an increase in PAM application rates.

**Table 2:** The amount ammonium nitrogen losses in runoff and sediments and the proportion of nitrogen loss forms for each PAM application rate under different rainfall intensities.

Designed rainfall intensity (mm h^−1^)	PAM (g·m^−2^)	Ammonium nitrogen losses in runoff(mg)	Ammonium nitrogen losses in sediments (mg)	Ammonium nitrogen losses from slope(mg)	The percentage of ammonium nitrogen losses in runoff (%)	The percentage of ammonium nitrogen losses in sediments (%)
50	0	131.34b	30.95d	162.28bc	80.93	19.07
	1	52.48e	9.24e	61.72d	85.03	14.97
	2	64.50d	5.70f	70.20d	91.88	8.12
	4	136.62b	4.46f	141.08c	96.84	3.16
80	0	182.99a	134.73a	317.72a	57.59	42.41
	1	139.77b	69.28b	209.04b	66.86	33.14
	2	88.57c	44.30c	132.87c	66.66	33.34
	4	170.35a	23.16d	193.51b	88.03	11.97

### Modeling ammonia nitrogen concentrations in runoff

Parameters of the developed model in this study can be obtained through different methods. The saturated moisture content and the bulk density were measured by the ring method. The saturated moisture content (${\theta }_{s}$) was 0.50 cm^3^·cm^−3^, and the bulk density (${\rho }_{s}$) was 1.45 g·cm^−3^. The ${t}_{p}$ and *S* are shown in Table 1, and the adsorption partition coefficients of different treatments are shown in Fig. S2. The adsorption partition coefficient of NH_4_^+^ increased with the PAM application rate. The convective mass transfer coefficient (${k}_{m}$) and the depth of the mixing layer (${h}_{m}$) were inversely estimated by fitting the NH_4_^+^ concentration data to the proposed solute transport model. The suitability of the curve fitting was quantified by *RMSE*, *R*^2^ and *NSE* measures.

The *RMSE*, *R*^2^*and NSE* results indicated that the process of NH_4_^+^ concentration in runoff over rainfall time for different treatments could be well described by the proposed runoff solute transport model. However, the *R*^2^ of 80 mm·h^−1^ rainfall intensity was smaller compared with the *R*^2^ of 50 mm·h^−1^ rainfall intensity. This indicated that the proposed runoff solute transport model was more suitable for small rainfall intensities. The convective mass transfer coefficient, ${k}_{m}$, mainly depends on the erosion rate and convective diffusion^9,10^. The erosion rate was considered a constant for the whole rainfall duration in the proposed model in this study. The large rainfall intensity easily caused gully erosion^47^, and the erosion rates were more unstable in a rainfall intensity of 80 mm·h^−1^ than that of 50 mm·h^−1^, which meant that the error between simulated value and actual value of ${{k}}_{m}$ was relatively small for 50 mm·h^−1^ compared with that of 80 mm·h^−1^. Therefore, the transport of solute was better described by this model for a 50 mm·h^−1^ rainfall intensity.

The values of ${{k}}_{m}$ in the solute transport model varied with rainfall intensity and PAM application rates. As shown in Table 3, the ${k}_{m}$ increased with an increase in rainfall intensity but increased first and then decreased with the increase in the PAM application rate. As indicated earlier, ${k}_{m}$ is mainly determined by erosion rates and convective diffusion. PAM application not only decreased the erosion rate but also increased the solutes convective diffusion period between runoff and soil solution. Therefore, the effect of PAM application on ${k}_{m}$ is a combination of erosion rate and convective diffusion.

**Table 3:** The ${k}_{m}$ and ${h}_{m}$ parameters obtained from the model and the*R*^2^, *RMSE* and *NSE* of the measured concentration and the simulated data.

Rainfall intensity (mm h^−1^)	PAM (g·m^−2^)	${k}_{m}$ (m·min^−1^)	${h}_{m}$ (mm)	*R* ^2^	*RMSE*	*NSE*
50	0	0.071	18.28	0.89	0.59	0.88
	1	0.055	13.35	0.99	0.11	0.99
	2	0.066	8.81	0.99	0.08	0.99
	4	0.079	7.85	0.95	0.37	0.94
80	0	0.079	21.38	0.85	0.30	0.85
	1	0.069	17.60	0.79	0.24	0.79
	2	0.056	12.65	0.90	0.15	0.90
	4	0.080	9.32	0.91	0.16	0.91

Further regression analysis was performed to investigate the relationships among ${k}_{m}$, rainfall intensity and PAM application rate. The regression equation can be express as follows:

13 $${k}_{m}=(0.0027{R}_{PAM}^{2}-0.01{R}_{PAM}+0.048){r}^{0.11}\,\,\,\,{R}^{2}=0.76$$

**Figure 5:**
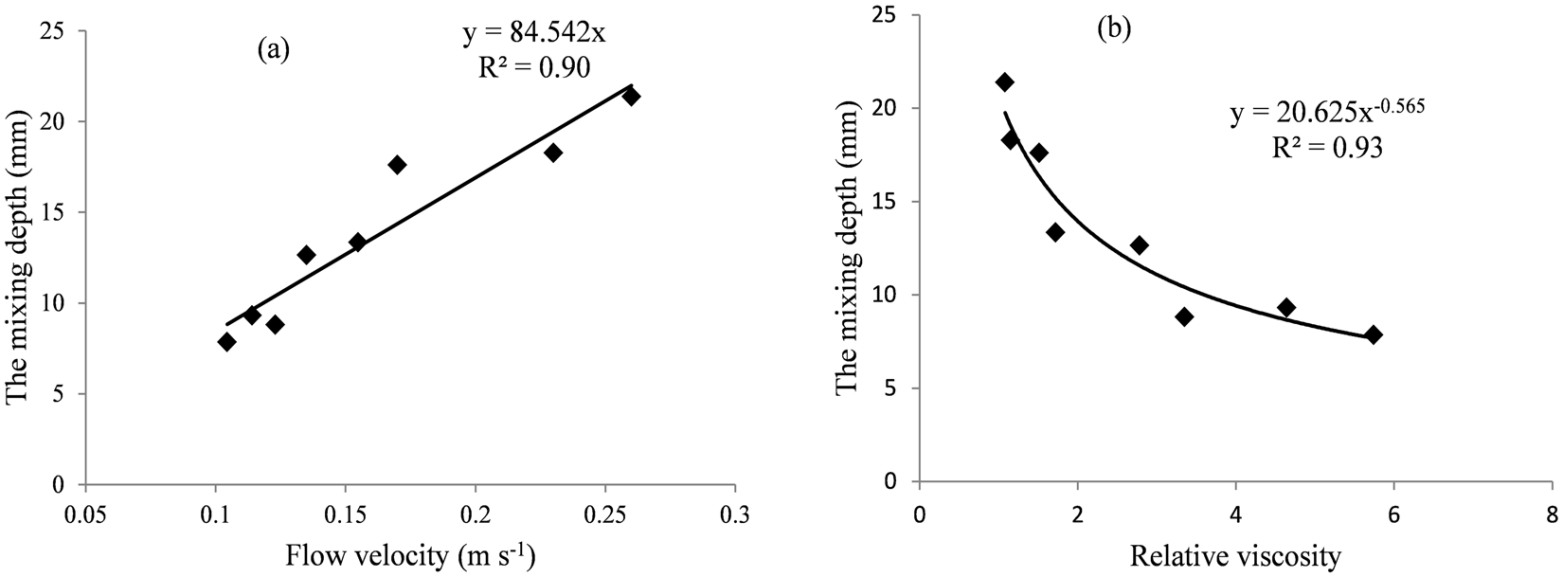
The mixing depth as a function of flow velocities and relative viscosity. Symbols = data; lines = fitted regressions.

The mixing depth, ${h}_{m}$, increased with an increase in rainfall intensity but decreased with an increase in PAM application rate (Table 3). Yang^17^ found similar observations through curve fitting of the mixing-depth model. As shown in Fig. 5, the mixing depth linearly increased with an increase in flow velocity, but it decreased exponentially with an increase in relative viscosity of runoff. The mixing depth had positive relationships with the erosion rate^4,17^. An increase in flow velocity would increase the flow energy^48^ which would increase the erosion rate, finally resulted in the increase of mixing depth.

The relationship between ${h}_{m}$ and rainfall intensity and the PAM application rate was further investigated by regression analysis. The regression equation was expressed as follows:

14 $${h}_{m}=2.66{e}^{-0.24{R}_{PAM}}{r}^{0.48}\,\,\,\,\,{R}^{2}=0.95$$

**Figure 6:**
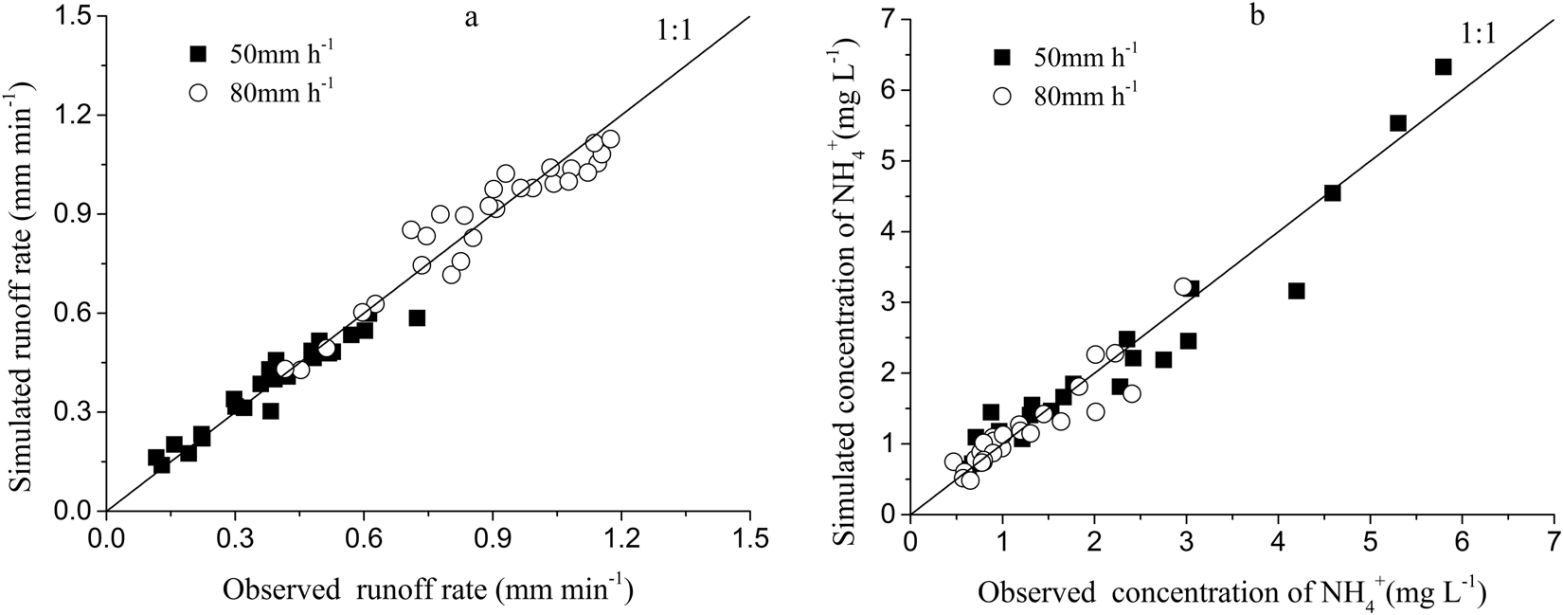
Simulated versus observed graphs for the runoff rate and the concentration of NH_4_^+^ in runoff.

The values of $S$, ${{k}}_{m}$ and ${h}_{m}$ can be calculated by Eqs (7), (13) and (14), respectively. After substituting $S$, ${{k}}_{m}$ and ${h}_{m}$ values into the proposed mathematical models, the runoff rates and the NH_4_^+^ concentrations in runoff with rainfall duration can then be calculated. As shown in Fig. 6, the simulated values are consistent with the observed values.

## Conclusions

In conclusion, the experiment showed the effects that PAM application had on runoff rate, flow velocity, erosion rate and ammonium transport processes from soil to runoff. To describe this process, a simple mathematical model was developed in this study. The runoff rate was increased with the increase in rainfall intensity but was first reduced and then increased with the PAM application rate. The runoff processes were well described by the runoff model based on Philip’s formula. The relationships between PAM application and model parameters, such as sorptivity (*S*), were expressed as quadratic functions. Flow velocity and erosion rates decreased with the increase in the PAM application rate. The NH_4_^+^ concentrations in runoff decreased with rainfall time; it was lower for PAM application treatments. The NH_4_^+^ transport with runoff can be well-described by the proposed solute transport model. Furthermore, the convective mass transfer coefficient (${k}_{m}$) and depth of the mixing layer (${h}_{m}$) increased as a power function with the increase in rainfall intensity. A quadratic function can be used to describe the relationship between the convective mass transfer coefficient and PAM application rate. However, the depth of the mixing layer exponentially decreased with the increase in the PAM application rate.

In summary, the proposed model in this study can accurately simulate the solute concentration in runoff. However, all experiments were conducted on a slope of 5° in the loess area. The practicability of the model for different areas, slope degrees and slope shapes needs to be further evaluated.

## Electronic supplementary material

Supplementary information
