# Supplementary material for: Mathematical model of ammonium nitrogen transport with overland flow on a slope after polyacrylamide application
Source: Sci Rep. 2018 Apr 23;8:6380. doi: 10.1038/s41598-018-24819-9 (PMC5913245; doi:10.1038/s41598-018-24819-9)
Supplement: Supplementary file 1 — Supplementary information [file 41598_2018_24819_MOESM1_ESM.pdf]

Mathematical model of ammonium nitrogen transport with overland flow on a slope after polyacrylamide application

Chang Ao[1], Peiling Yang[1*], Shumei Ren[1], Weimin Xing[1]

[1] College of Water Conservancy and Civil Engineering, China Agricultural University, Beijing, 100083, China

*Correspondence: cau_yangpeiling @163.com

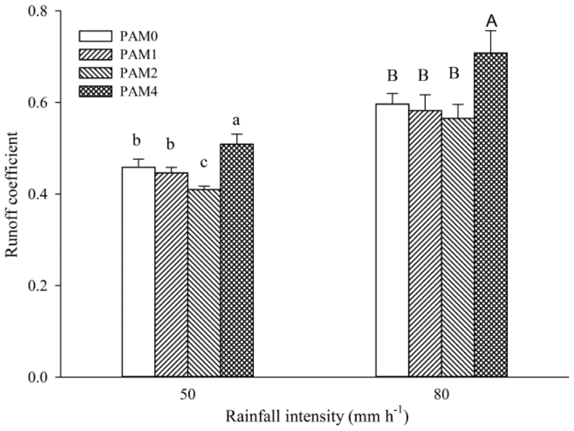

Figure S1. Runoff coefficients of different PAM application rates under rainfall intensities of 50 and 80 mm h^{-1}. Columns labeled with different letters within groups indicate significant differences at p < 0.05. Error bars in figure refer to standard deviation.

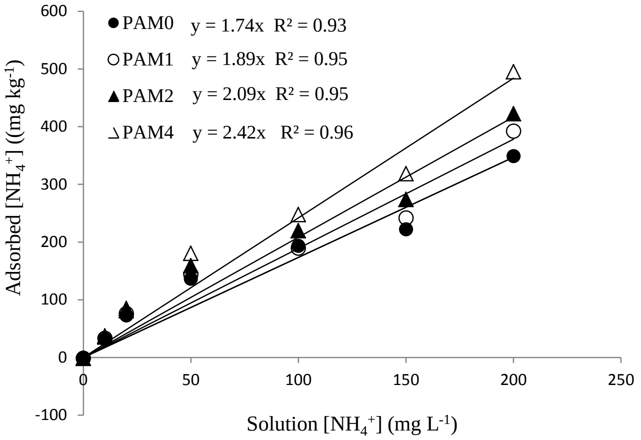

Figure S2. NH_4^+ adsorption isotherm. Symbols = data; lines = fitted regressions.

The adsorbed capacities of NH_4^+, as a function of the equilibrium concentration, are shown in Fig. 6. The adsorbed capacities of NH_4^+ in soil increased with the increase in equilibrium solution concentration. To determine the NH_4^+ adsorption parameters, several studies were made to fit the experimental data to the Freundlich equation and the Langmuir equation. Many studies have found that the adsorption partition coefficients can be estimated by the tangent to the Langmuir isotherm. In the Langmuir isotherm equation, the initial function is very close to the linear partition relationship. Therefore, a simple linear regression of the NH_4^+ adsorption isotherm was employed to approximate the NH_4^+ adsorption partition coefficient (Fig. 6). The adsorption partition coefficient of NH_4^+ increased with the increase in the PAM application rate. The anionic charges

on PAM may adsorb the positively charged nutrient ions from soil, water or excluded anions. In this study, anionic PAM was selected as the testing parameter. Thus, the PAM application increased the adsorbed capacities of NH_4^+ compared with no PAM application.
